# Surface disinfection and protective masks for SARS‐CoV‐2 and other respiratory viruses: A review by SIdP COVID‐19 task force

**DOI:** 10.1111/odi.13646

**Published:** 2020-10-06

**Authors:** Luigi Barbato, Francesco Bernardelli, Giovanni Braga, Marco Clementini, Claudio Di Gioia, Crisitnano Littarru, Francesco Oreglia, Mario Raspini, Eugenio Brambilla, Ivo Iavicoli, Vilma Pinchi, Luca Landi, Nicola Marco Sforza, Raffaele Cavalcanti, Alessandro Crea, Francesco Cairo

**Affiliations:** ^1^ Research Unit in Periodontology and Periodontal Medicine Department of Department of Experimental and Clinical Medicine University of Florence Florence Italy; ^2^ Private practice Modena Italy; ^3^ Private practice Tricesimo(Udine) Italy; ^4^ Department of Periodontology Università Vita‐Salute San Raffaele Milano Italy; ^5^ Private practice Bari Italy; ^6^ Private practice Roma Italy; ^7^ Private practice Verona Italy; ^8^ Private practice Cesena Italy; ^9^ Department of Biomedical Surgical and Dental Sciences University of Milan Milan Italy; ^10^ Section of Occupational Medicine Department of Public Health University of Naples Federico II Naples Italy; ^11^ Department of Health Sciences Section of Medical Forensic Sciences University of Florence Florence Italy; ^12^ Private Practice Rome Italy; ^13^ SIdP Florence Italy; ^14^ Private Practice Bologna Italy; ^15^ Private Practice Bari Italy; ^16^ Private Practice Viterbo Italy; ^17^ Research Unit in Periodontology and Periodontal Medicine Department of Experimental and Clinical Medicine University of Florence Florence Italy

**Keywords:** COVID‐19, Personal protection equipment, SARS‐CoV‐2, Surface disinfection

## Abstract

**Objectives:**

Primary focused question for this systematic review (SR) was *“Which is the evidence about surfaces decontamination and protection masks for SARS‐Cov‐2 in dental practice?”* Secondary question was “*Which is the evidence about surfaces decontamination and protection masks against airborne pathogens and directly transmitted viral pathogens causing respiratory infections?”*

**Materials and Methods:**

PRISMA guidelines were used. Studies on surface decontamination and protective masks for SARS‐CoV‐2 in dental practice were considered. Studies on other respiratory viruses were considered for the secondary question.

**Results:**

No studies are available for SARS‐CoV‐2. Four studies on surface disinfection against respiratory viruses were included. Ethanol 70% and sodium hypochlorite 0,5% seem to be effective in reducing infectivity by > 3log TCID. Four RCTs compared different types of masks on HCW. The single studies reported no difference for laboratory‐diagnosed influenza, laboratory‐diagnosed respiratory infection, and influenza‐like illness. A meta‐analysis was not considered appropriate.

**Conclusions:**

There is lack of evidence on the efficacy of surface disinfection and protective masks to reduce the spread of SARS‐CoV‐2 or other respiratory viruses in dentistry. However, the consistent use of respirator and routine surface disinfection is strongly suggested. There is urgent need of data on the efficacy of specific protection protocols for dental HCW against viral infections.

## 1 INTRODUCTION

On January 7, 2020, a novel coronavirus called 2019‐nCov was identified in patients affected by pneumonia of unknown etiology in Wuhan, China. The virus was renamed SARS‐CoV‐2, and the clinical disease, COVID‐19. On August 2020, more than 23.4 million of people were affected all over the word. Healthcare workers (HCWs) account for a significant proportion of infections (Chou et al., 2020). On August, 30,415 cases and 94 deaths between HCWs were confirmed accounting for 11.9% of all Italian cases (www.epicentro.iss/coronavirus).

The principal route of transmission for the SARS‐CoV‐2 is direct contact with respiratory droplets (>5–10 μm), and the other route is indirect contact through fomites (Chan et al., 2020; Li et al., 2020a). The airborne transmission (droplet nuclei, <5–10 μm) is possible but not demonstrated (Meselson, 2020), even if the viral RNA was found in the aerosol of different hospital areas (Liu et al., 2020). The transmission routes are similar to other respiratory viruses (e.g., SARS‐CoV, MERS).

In general, respiratory droplets represent a direct source of infection for respiratory viruses and also rapidly fall creating fomites near the infected subjects (<1 m). Contrary droplet nuclei may remain in the air for a long period and could be inhaled, thus potentially represent a source of infection at greater distance (>1 m). The SARS‐CoV‐2 could remain viable in the aerosol for hours (Lednicky et al., 2020; Doremalen et al., 2020) and infected droplets could precipitate, thus contaminating the operative surfaces (Guo et al., 2020).

Therefore, in a dental setting, HCWs are exposed to infection risk through direct contact with respiratory droplets, but also through indirect contact with contaminated surfaces or instruments (Ionescu et al., 2020; Peng et al., 2020; Zemouri, et al., 2020). Additionally, dental HCWs are exposed also to airborne produced during the usually performed aerosol‐generating procedures (AGPs). This observation raised a debate on airborne transmission for SARS‐CoV‐2 and other respiratory viruses in a dental setting.

Although dental HCWs could be considered at higher risk of respiratory infections due to the characteristic of the dental setting and the performed procedures, no conclusive data are available demonstrating the increased risk (Samaranayake & Peiris, 2004).

The surface decontamination procedures alongside the use of personal protective equipment (PPE), including protective mask, are effective in reducing the infection among HCW, especially during outbreaks (Verbeek et al., 2020). These approaches for infection control are routinely used in the dental practice, but the evaluation of their efficacy during SARS‐CoV‐2 spread should be examined. The appropriate implementation of PPE and disinfection procedures raises relevant medico‐legal issues for dental professionals and legal challenges for authorities deputed to provide guidance on correct use and adequate supplies (Dyer, 2020).

The primary aim was to review the evidence about surface disinfection and protection mask usage in dental practice for SARS‐CoV‐2. Due to lack of evidence, we also add a secondary aim to review the evidence for other directly transmitted viral pathogens that cause respiratory infections.

## 2 MATERIALS AND METHODS

### 2.1 Protocol and focused question

The protocol for this SR was prepared according to PRISMA guidelines (Hutton et al., 2015). The focused question was *“Which is the evidence about surfaces decontamination and protection masks for SARS‐Cov‐2 in dental practice?”*

The secondary focused question was “*Which is the evidence about surfaces decontamination and protection masks against airborne pathogens and directly transmitted viral pathogens causing respiratory infections?”*

### 2.2 Eligibility criteria and information sources

All the studies reporting evidence regarding the efficacy of surface decontamination procedures and protective mask usage for SARS‐CoV‐2 in dental practice were considered. Only English‐language manuscripts were included. Searches were conducted on PubMed and Embase on 24, August 2020. Additionally, the Cochrane special section for COVID‐19 and the references of the included studies were checked also. Full‐text assessment of all the articles on COVID‐19 published on dental journals or about dental procedures was performed. For details regarding search strategy, study selection, and data collection process, see supporting information (Appendix S1).

As this search did not provide studies on SARS‐CoV‐2 in dentistry, we reviewed literature on other viral pathogens causing respiratory infections using a specific search strategy (Appendix S1). All the studies comparing the efficacy of different disinfection agents on inanimate surfaces or using carrier test in terms of viral load reduction/inactivation were considered.

Randomized clinical trials comparing the efficacy of different protective masks in preventing respiratory infections among HCWs in terms of laboratory‐confirmed infection were also included. The references of previous SR and included studies were checked also for additional titles. Only English‐language manuscripts were included.

## 3 RESULTS

The search for surface disinfection and protective mask usage in dental practice for SARS‐CoV‐2 yields 8,749 titles; however, no study was eligible for inclusion. (For detailed information see Appendix S2 and Appendix S3.) None of the retrieved studies reported original data on surface decontamination and protection mask usage in dental practice for SARS‐CoV‐2.

The secondary search on surface disinfection and protective masks for other viral pathogens causing respiratory infections yielded 1524 titles. During the screening of title and abstract, 1502 titles were excluded. Out of 22 studies evaluated full text, 14 were excluded with reason, while 4 studies on surface disinfection (Becker et al., 2017; Jeong et al., 2010; Rabenau et al., 2014; Sattar et al., 1989) and 4 RCTs on protective masks (Loeb et al., 2009; MacIntyre et al., 2011; MacIntyre et al., 2013; Radonovich et al., 2019) were included. (For detailed information, see Appendix S4 and Appendix S5.)

The four studies on surface disinfection against respiratory viruses reported the efficacy of different disinfectant agents in carrier test in terms of viral titer reduction expressed as virus log10 reduction factor for tissue culture infective dose 50 (TCID 50). A reduction factor >3log TCID 50/ml is regarded as evidence of virucidal activity (inactivation ≥ 99.99%). (For details, see Table 1.) Becker et al. reported that peracetic acid PPA ≥ 400 ppm applied for 5 min is effective against adenovirus (Becker et al., 2017). Rabenau et al. reported that glutaraldehyde (GDA) 125ppm, ethanol 55%, 1‐propanol 30%, or higher concentrations applied for 5 min are effective against type 5 adenovirus (Rabenau et al., 2014). Ethanol 70%, sodium hypochlorite 0.5% or 1.0%, GDA 2%, and chloramine T 0.3% or 0.5% applied for 1 min were effective against type 5 adenovirus in the study of Sattar et al. (1989). The same study also showed that ethanol 70%; sodium hypochlorite 0.1% or 0.5%; GDA 2%; and chloramine T 0.1% or 0.3% had virucidal activity against HCov 229E, while ethanol 70%; sodium hypochlorite 0,1% or 0,5%; GDA 2%; chloramine T 0.01%, 0.1%, or 0.3%; and povidone‐iodine 10% were effective against type 3 parainfluenza virus. Jeong et al reported that ethanol 70% applied for 1 min on plastic coupon is effective against influenza A H1N1 virus (Jeong et al., 2010). The heterogeneity among experimental conditions in the included studies (i.e., type of viruses, type of carrier, exposure time) did not allow a meta‐analysis.

**Table 1:** Studies reporting the efficacy of different disinfectants on surfaces in terms of viral titer reduction expressed as virus log10 reduction factor TCID 50

Author	Test	Virus	Carrier	Agents	Time	Volume	Reduction factor
Becker et al., 2017	Carrier test EN17111:2017	Adenovirus	Sandblasted frosted glass	PAA 400ppm	5 min	50 μl	>4log TCID 50/ml
Rabenau et al., 2014, ^a^	Carrier test	Type 5 adenovirus	Stainless disk	GDA 125, 500, 1,000, 2000, 2,500 ppm; PPA 200, 500, 1,000, 1,500 ppm; ethanol 55, 60%, 1‐propanol 30%, 40%, 50%, 60%	5 min	50 μl	>4log TCID 50/ml
				Ethanol 40, 50%; 1‐propanol 10, 20%; 2‐propanol 40%, 60%	5 min	50 μl	<2 log TCID 50/ml
Jeong et al., 2010	Inanimate surface	Influenza A H1N1	Plastic coupon	0,1 mol/L NaOH; ethanol 70%	1min	2.0 ml	>2.78 log TCID 50/ml
				1‐propanol 70%	1 min	2.0 ml	3,70 log TCID 50/ml
Sattar et al., 1989	Carrier test	HCov 229E	Stainless steel	Ethanol 70%; sodium hypochlorite 0,1%, 0,5%; GDA 2%; chloramine T 0,1%.0,3%	1 min	20 μl	>3 log TCID 50/ml
				Benzalkoniumchloride 0,04; sodium hypochlorite 0,01%; chloramine T 0,01%; povidone‐iodine 10%	1 min	20 μl	<3 log TCID 50/ml
	Carrier test	Type 3 parainfluenza virus	Stainless steel	Ethanol 70%; sodium hypochlorite 0,1%, 0,5%; GDA 2%; chloramine T 0,01%, 0,1%, 0,3%; povidone‐iodine 10%	1 min	20 μl	>3 log TCID 50/ml
				Benzalkoniumchloride 0,04; sodium hypochlorite 0,01%	1 min	20 μl	<3 log TCID 50/ml
	Carrier test	Type 5 adenovirus	Stainless steel	Ethanol 70%; sodium hypochlorite 0,5%, 1,0%; GDA 2%; chloramine T 0,3%.0,5%	1 min	20 μl	>3 log TCID 50/ml
				Benzalkoniumchloride 0,04; sodium hypochlorite 0,01, 0.1%; chloramine T 0,01%, 0,1%, povidone‐iodine 10%	1 min	20 μl	<3 log TCID 50/ml

Abbreviations: GDA, glutaraldehyde; HCov, human coronavirus; PAA, peracetic acid; TCID, tissue culture infectious dose.

^a^ This was a multilaboratory trial, and we reported data of the laboratory number 2 because they had less missing data than the other laboratories in the study.

For protective mask usage against other respiratory viral infections, 4 RCTs were included (Loeb et al., 2009; MacIntyre et al., 2011; MacIntyre et al., 2013; Radonovich et al., 2019). All the studies were in hospital settings and enrolled HCWs. The included studies tested the efficacy of different masks (surgical mask vs. N95 respirators) in terms of laboratory‐confirmed influenza, laboratory‐confirmed other respiratory viruses, and influenza‐like illness (ILI). The data from single studies showed no statistically significant difference for the aforementioned outcomes comparing surgical mask and N95 fit‐tested respirators. (For detailed information, see Table 2.) MacIntyre et al. reported data on clinical respiratory illness (CRI) in two RCTs showing conflicting results. While in the first RCT (MacIntyre et al., 2011), there was no difference in terms of CRI between surgical mask and N95 fit‐tested respirators, and in the second RCT (MacIntyre et al., 2013), there was a statistically significant difference favouring N95 respirators. Although there were similar outcomes in the included studies, a meta‐analysis was not considered appropriate due to heterogeneity among setting, mask type, fit testing, and outcome measurements.

**Table 2:** RCTs testing the efficacy of different protective masks in term of laboratory‐confirmed influenza, ILI, laboratory‐confirmed other respiratory viruses and CRI

Author	Participants		Medical mask	N95 fit tested	N95 not fit tested	N95 fit tested targeted use	
Loeb et al., 2009	442 nurses 8 Hospitals	Laboratory‐confirmed influenza	50/212 (23.6%)	48/210 (22.9%)			Absolute risk difference: −0.73%; 95% CI [−8.8 to 7.3]; *p* = .86
		Laboratory‐confirmed other respiratory viruses	20/212 (9.4%)	22/210 (10.5%)			Absolute risk difference: 1.04%; 95% CI [−4.67 to 6.76]; *p* = .72
		ILI	9/212 (4.2%)	2/210 (1.0%)			Absolute risk difference: −3.29%; 95% CI [−6.31 to 0.28]; *p* = .6
MacIntyre et al., 2011	1,441 HCW 15 hospitals	Laboratory‐confirmed influenza	5/492 (1%)	3/461 (0,7%)	0/488 (0%)		OR N95 fit‐tested versus medical mask: 0.64; 95% CI [0.15 to 2.68]; *p* = .54
		Laboratory‐confirmed other respiratory viruses	13/492 (2.6%)	8/461 (1.7%)	5/488 (1%)		OR N95 fit‐tested versus medical mask: 0.69; 95% CI [0.24 to 2.03]; *p* = .5
		ILI	3/492 (0.6%)	1/461 (0,2%)	2/488 (0.4%)		OR N95 fit‐tested versus medical mask: 0.35; 95% CI [0.04 to 3.42]; *p* = .37
		CRI	33/492 (6.7%)	21/461 (4.6%)	16/488 (3.3%)		OR N95 fit‐tested versus medical mask: 0.76; 95% CI [0.27 to 2.13]; *p* = .6
MacIntyre et al., 2013	1669 HCW 19 Hospitals	Laboratory‐confirmed influenza	1/572 (0.2%)	3/581 (0.5%)		2/516 (0.4%)	*p* = .3241
		Laboratory‐confirmed other respiratory viruses	19/572 (3.3%)	13/581 (2.2%)		17/516 (3.3%)	*p* = .4394
		ILI	4/572 (0.7%)	6/581 (1.0%)		2/516 (0.4%)	*p* = .5416
		CRI	98/572 (17.1%)	42/581 (7.2%)		61/516 (11.8%)	***p* = .0238**
Radonovich et al., 2019	2,862 HCW^a^	Laboratory‐confirmed influenza	207/2512 (8.2%)	193/2668 (7.2%)			Difference: 1.0% per 1,000 HCW season 95% CI [−0.5 to 2.5]; *p* = .18
		Laboratory‐confirmed other respiratory viruses	371/2512 (14.8%)	417/2668 (15.2%)			Difference: −8.6%, per 1,000 HCW season 95% CI [−28.2 to 10.9]; *p* = .39
		ILI	128/2512 (8.2%)	166/2668 (7.2%)			Difference: −11.3%, per 1,000 HCW season 95% CI [−28.2 to 10.9]; *p* = .08

Abbreviations: CRI, clinical respiratory illness; HCW, healthcare worker; ILI, influenza‐like illness; OR, odds ratio.

^a^ Out of 2,868 HCWs, 1,416 participated more than 1 year for a total of 5,180 HCW season.

## 4 DISCUSSION

During the last months, a fast COVID‐19 outbreak was registered worldwide. Epidemiologic data suggest that HCWs are at risk of infection for SARS‐CoV‐2. In Italy, HCWs accounted for the 12.2% of all COVID‐19 cases on July 2020 (www.epicentro.iss/coronavirus). In order to avoid the spread of COVID‐19 due to dental treatments, therapies were restricted to emergencies and were performed adhering to strict clinical recommendations suggested by Italian Ministry of Health, Dental Societies and Associations (https://portale.fnomceo.it; https://www.andi.it; https://www.sidp.it).

Dental HCWs are in close contact with patient mouths, very frequently performing AGPs (i.e., sonic/ultrasonic device). The SARS‐CoV‐2 RNA was found in the saliva of infected patients (To et al., 2020; Zhang et al., 2020); thus, saliva could be a source of infection (Xu et al., 2020). The aerosols generated during an AGP are mixed with patient saliva/blood and is contaminated by bacteria and viruses (Cleveland et al., 2016; Harrel & Molinari, 2004) acting thus as a carrier of infection (Ionescu et al., 2020; Zemouri et al., 2017; Zemouri, et al., 2020). Therefore, dental HCWs and patients attending dental procedures could be considered potentially at risk of infections due to direct contact with respiratory droplets, indirect contact with fomites, and inhalation of droplet nuclei.

In a clinical perspective, the identification of COVID‐19 patients is a key factor in dental settings. Although the telephonic triage has been suggested to potentially screen positive patients, two possible scenarios may be identified. The first, as suggested by epidemiology reports on COVID‐19 (Li et al., 2020a), is a contagious asymptomatic patient requiring dental treatments, not yet diagnosed for SARS‐CoV‐2. Under these conditions, the use of PPE appears critical to reduce the possible risk of infection (Dugré et al., 2020; Zemouri, et al., 2020). The other scenario includes a patient with diagnosed active COVID‐19 requiring urgent therapy: In these circumstances, dental treatments should be performed only in hospital setting where a specific management of COVID‐19 patients is possible.

A recent publication summarizes the infection control measures in dental health care for SARS‐CoV‐2. These measures have a hierarchy of effectiveness, intervening at different levels. Measures acting on the source of the virus are generally more effective than measures applied to the HCW (Volgenant et al., 2020). Within this context, the procedure of surface disinfection acts eliminating the secondary virus reservoir, while the use of protective masks/PPE protects the dental HCW. The first aim of this SR was to assess the efficacy of surface decontamination procedures and protective mask usage in dental practice for SARS‐CoV‐2. Unfortunately, no direct evidence data were available to answer the focused question on decontamination and masks in dental setting. A recent experiment demonstrated that SARS‐CoV‐2 could remain viable on different surfaces. The virus was more stable on stainless steel and plastic, less on cardboard, and was find viable up to 72 hr on these surfaces (Doremalen et al., 2020) so representing a potential source of infection. This observation introduces an urgent need of studies on disinfection agents and SARS‐CoV‐2.

The secondary focused question was on the use of surface disinfection and protective masks to protect against airborne pathogens and directly transmitted viral pathogens that cause respiratory infections. Evidence of biocidal agents against other coronaviruses could be retrieved from a recent systematic review. In carrier tests, ethanol (70%), sodium hypochlorite (0,1%), and glutaraldehyde (2%) for 1 min were effective to reduce endemic human coronavirus (HCoV) infectivity by > 3log TCID 50/ml in suspension tests, and ethanol (78%–95%), 2‐propanol (70%–100%), glutaraldehyde (0.5%–2.5%), formaldehyde (0.7%–1%), and povidone‐iodine (0.23%–1%) reduced SARS‐CoV infectivity by > 3log TCID 50/ml (Kampf, 2020b; Kampf et al., 2020a).

In our review, suspension test studies were not considered. We evaluated only carrier test or test on inanimate surface because these better simulate a real clinical setting. Only four studies testing different disinfectants on different surfaces (glass, plastic, stainless) were included. Data from single studies suggest that ethanol 70%, sodium hypochlorite 0,5%, and GDA 2% were effective in reducing the viral titer of > 3log TCID 50/ml for type 5 adenovirus, HCov 229E, and type 3 parainfluenza virus (Becker et al., 2017; Jeong et al., 2010; Rabenau et al., 2014; Sattar et al., 1989).

Very recently, European Centre for Disease Prevention and Control (ECDC) suggested to use 0.05% sodium hypochlorite or 70% ethanol for surface disinfection in a healthcare setting (ECDC, 2020). The use of 0.05% sodium hypochlorite instead of higher concentrations was suggested by ECDC to reduce irritant effects on the mucosae. It is mandatory to consider that glutaraldehyde usage as disinfectant agent is not allowed in most European countries, and it should be kept in mind that chronic glutaraldehyde utilization may expose to important side effects, including sensitization of skin and respiratory diseases, and a potential carcinogenic activity (Takigawa & Endo, 2006). Considering all these elements, ethanol 70% or sodium hypochlorite 0.05% could be suggested for surface disinfection.

Four RCTs comparing the efficacy of different masks against other respiratory viruses were included (Loeb et al., 2009; MacIntyre et al., 2011; MacIntyre et al., 2013; Radonovich et al., 2019). All the included studies compared surgical mask versus N95 fit‐tested respirators in HCWs. In a RCT, not‐fit‐tested N95 was used also, while another RCT proposed a targeted use of fit‐tested N95 respirator. Even if data are scanty and controversial, the reported outcomes in the single studies provided a trend of similar efficacy in terms of laboratory‐diagnosed influenza, laboratory‐diagnosed respiratory viral infections, and ILI for surgical mask versus fit‐tested N95 respirator. Data regarding the diagnosis of clinical respiratory illness (CRI) are controversial. A RCT suggest the use of N95 respirators in preventing CRI (MacIntyre et al., 2013), while another not reported difference among surgical masks and respirators (MacIntyre et al., 2011). However, CRI could be bacteria‐related and not virus‐related and this might be not properly explored. All the included studies are performed also in hospital settings but no included trial has a proper control group to monitor the infection source outside, thus limiting the possibility to extend these findings to dental setting.

The rationale to use a filtering facepiece respirators (such as N95, KN95, and FFP2), rather than a surgical mask, is also due to the higher capability in protection against the small aerosol particles (<1 μm) (Bałazy et al., 2006; Qian et al., 1998) produced during dental AGPs. A filtering facepiece respirator has to be sealed properly to be protective, so better protection is valuable when a leak‐test is performed. If there is not a peripheral seal, the airborne could leak around the edges of the respirator. On this way, the Respiratory Protection Standards (1910.134) settled by the US Occupational Safety and Health Administration (OSHA) require a fit test to identify the right model and size of respirator for each worker, an annual fit test to maintain the expected level of protection and a user seal check each time the worker put on a respirator. Other countries have different policies. Additionally, the use of filtering facepiece respirators requires specific face‐to‐face training (Verbeek et al., 2020). MacIntyre et al. and Radonovic et al. used a qualitative fit test, while in the study of Loeb et al. the participants provided a current fit‐test certification. However, none of included studies reported a quantitative leak test, and this could have hindered the difference between surgical masks and N95 respirators.

The included RCTs failed to find differences between HCWs randomized to fit‐tested N95 respirator group or surgical mask group in terms of laboratory‐diagnosed infections. Interestingly, infections range from 1.7% to 15.2% and were reported in almost all respirators and surgical mask groups. Surprisingly, no infection in the N95 not‐fit‐test group in the study of McIntyre et al. was reported (Loeb et al., 2009; MacIntyre et al., 2011; Radonovich et al., 2019). These differences could be explained by different experimental condition, setting, locations, and the lack of a proper fit test. Furthermore, respirators are uncomfortable to wear and it is difficult to be compliant with for prolonged time (Jefferson et al., 2011), and this could have reduced the protective efficacy hindering the differences with the surgical masks. From the overall assessment of the evidence, the use of respirators for dental HCWs seems to be indicated in protecting against respiratory viruses, since dental office is a specific medical setting in which HCWs are very often exposed to potentially infected aerosol.

It should be keep also into account that availability of filtering facepiece respirators may be difficult during an outbreak. The possible disinfection of these respirators applying different procedures, including ionized hydrogen peroxide (Cheng et al., 2020) or ultraviolet C light (Cadnum et al., 2020) or combinations (Bergman et al., 2010), has been proposed. However, reported outcomes are controversial and heterogeneous, thus suggesting caution in terms of routine applicability.

It is mandatory to underline that protective mask usage has to be considered alongside other PPE (i.e., gowns, gloves). The whole protocol appears more important than the single protective item.

Although is difficult to retrieve specific information in dental literature (Li et al., 2020b; Volgenant et al., 2020), the importance of PPE during an outbreak has been described in case–control and retrospective cohort studies, underlying the importance in using masks, gowns, and gloves for reducing risk of infection compared with their inconsistent use (Verbeek et al., 2020). Unfortunately, it is not possible to clearly assess which is the best PPE procedure or the perfect combination. The majority of the available information is related to laboratory simulative studies useful for setting the equipment physical standard requirements. However, the experimental condition of these simulations could be really different from the clinical settings. The aforementioned limitations in testing strongly reduce the possible generalizability of present information. Based on the previous evaluation, WHO recommended wearing gloves, masks, goggles, face shields, and long‐sleeved gowns adding a filtering facepiece respirator only during an AGP on a COVID‐19‐positive patient (WHO, 2020).

## 5 CONCLUSIONS AND RECOMMENDATIONS

- No direct evidence is available for surface disinfection and protective masks for SARS‐CoV‐2 or other respiratory viruses in dental setting.
- Although direct evidence is missing, application of ethanol 70% or sodium hypochlorite 0,5% for 1 min should be considered effective to reduce SARS‐CoV‐2 or respiratory virus infectivity over surfaces.
- Surgical masks may be not adequate to prevent respiratory virus transmission to dental HCWs.
- Although limited in terms of consistency, evidence showed that a proper use of filtering facepiece respirators should be recommended especially performing an AGP.
- There is the urgent need to test efficacy of specific protection protocols for dental HCWs for SARS‐CoV‐2 and other respiratory viruses.

## CONFLICT OF INTEREST

The authors have nothing to disclose.

## AUTHOR CONTRIBUTIONS

**Luigi Barbato:** Conceptualization; Writing‐original draft. **Francesco Bernardelli:** Resources. **Giovanni Braga:** Data curation. **Marco Clementini:** Investigation. **Claudio Di Gioia:** Data curation. **Crisitiano Littarru:** Methodology. **Francesco Oreglia:** Methodology. **Mario Raspini:** Formal analysis. **Eugenio Brambilla:** Writing‐review & editing. **Ivo Iavicoli:** Writing‐original draft. **Vilma Pinchi:** Writing‐original draft. **Luca Landi:** Writing‐review & editing. **Nicola Marco Sforza:** Writing‐review & editing. **Raffaele Cavalcanti:** Data curation; Writing‐review & editing. **Alessandro Crea:** Data curation. **Francesco Cairo:** Conceptualization; Validation; Writing‐original draft.

### PEER REVIEW

The peer review history for this article is available at https://publons.com/publon/10.1111/odi.13646.

## Supporting information

Appendix S1

Appendix S2

Appendix S3

Appendix S4

Appendix S5
