# Supplementary material for: Surface disinfection and protective masks for SARS‐CoV‐2 and other respiratory viruses: A review by SIdP COVID‐19 task force
Source: Oral Dis. 2020 Oct 6:10.1111/odi.13646. Online ahead of print. doi: 10.1111/odi.13646 (PMC7646272; doi:10.1111/odi.13646)
Supplement: Supplementary file 1 — Appendix S1 [file ODI-9999-0-s004.docx]

**Appendix 1: Search strategy, Studies selection, Data collection process**

***Search strategy for SARS-Cov-2 and dentistry***

On-line searches were conducted on PubMed and Embase using the follow search strategies.

Pubmed search strategy (24/08/2020): 6303 titles

("COVID-19"[All Fields] OR "COVID-2019"[All Fields] OR "severe acute respiratory syndrome coronavirus 2"[Supplementary Concept] OR "severe acute respiratory syndrome coronavirus 2"[All Fields] OR "2019-nCoV"[All Fields] OR "SARS-CoV-2"[All Fields] OR "2019nCoV"[All Fields] OR (("Wuhan"[All Fields] AND ("coronavirus"[MeSH Terms] OR "coronavirus"[All Fields])) AND (2019/12[PDAT] OR 2020[PDAT]))) AND (("dental health services"[MeSH Terms] OR ("dental"[All Fields] AND "health"[All Fields] AND "services"[All Fields]) OR "dental health services"[All Fields] OR "dental"[All Fields]) OR ("aerosols"[MeSH Terms] OR "aerosols"[All Fields] OR "aerosol"[All Fields]) OR ("disinfection"[MeSH Terms] OR "disinfection"[All Fields]) OR protection[All Fields] OR mask[All Fields] OR surface[All Fields] OR "filter"[All Fields] OR "air conditioning"[All Fields] OR HEPA[All Fields] OR "air disinfection"[All Fields] OR "PPE"[All Fields] OR "personal protective equipment"[All Fields])

Embase search strategy (24/08/2020): 6721 titles

('covid 19'/exp OR 'covid 19' OR 'sars-cov-2' OR '2019ncov') AND ('dental'/exp OR dental OR 'dentistry'/exp OR dentistry OR 'aerosol'/exp OR aerosol OR 'disinfection'/exp OR disinfection OR 'protection'/exp OR protection OR 'surface'/exp OR surface OR 'mask'/exp OR mask OR 'air conditioning'/exp OR 'air conditioning' OR 'filter'/exp OR filter OR 'hepa' OR hepa OR 'ppe' OR 'personal protective equipment'/exp OR 'personal protective equipment')

Additionally, the Cochrane special section for Covid-19, the references of the included studies were checked and the Italian (ISS), European (ECDC), American (CDC) and global (WHO) recommendations were considered for additional information.

***Search strategy for other viruses***

On-line searches were conducted on PubMed, Embase and Cochrane using the following search strategies.

Pubmed search strategy (24/08/2020): 159 titles

(((((((("virology"[MeSH Subheading] OR "virology"[All Fields]) OR "viruses"[All Fields]) OR "viruses"[MeSH Terms]) OR "virus s"[All Fields]) OR "viruse"[All Fields]) OR "virus"[All Fields]) OR (((((("influenza s"[All Fields] OR "influenza, human"[MeSH Terms]) OR ("influenza"[All Fields] AND "human"[All Fields])) OR "human influenza"[All Fields]) OR "influenza"[All Fields]) OR "influenzae"[All Fields]) OR "influenzas"[All Fields])) OR "airborne pathogens"[All Fields]) AND (((((((((((((("disinfect"[All Fields] OR "disinfectable"[All Fields]) OR "disinfectants"[Pharmacological Action]) OR "disinfectants"[MeSH Terms]) OR "disinfectants"[All Fields]) OR "disinfectant"[All Fields]) OR "disinfected"[All Fields]) OR "disinfecting"[All Fields]) OR "disinfection"[MeSH Terms]) OR "disinfection"[All Fields]) OR "disinfections"[All Fields]) OR "disinfective"[All Fields]) OR "disinfects"[All Fields]) OR (((((((((((("disinfect"[All Fields] OR "disinfectable"[All Fields]) OR "disinfectants"[Pharmacological Action]) OR "disinfectants"[MeSH Terms]) OR "disinfectants"[All Fields]) OR "disinfectant"[All Fields]) OR "disinfected"[All Fields]) OR "disinfecting"[All Fields]) OR "disinfection"[MeSH Terms]) OR "disinfection"[All Fields]) OR "disinfections"[All Fields]) OR "disinfective"[All Fields]) OR "disinfects"[All Fields])) OR "biocidal agents"[All Fields])

FILTERS: randomized clinical trial; meta-analyses; systematic review; clinical trial

Pubmed search strategy (24/08/2020): 126 titles

(((((((("virology"[MeSH Subheading] OR "virology"[All Fields]) OR "viruses"[All Fields]) OR "viruses"[MeSH Terms]) OR "virus s"[All Fields]) OR "viruse"[All Fields]) OR "virus"[All Fields]) OR (((((("influenza s"[All Fields] OR "influenza, human"[MeSH Terms]) OR ("influenza"[All Fields] AND "human"[All Fields])) OR "human influenza"[All Fields]) OR "influenza"[All Fields]) OR "influenzae"[All Fields]) OR "influenzas"[All Fields])) OR "airborne pathogens"[All Fields]) AND (((("masks"[MeSH Terms] OR "masks"[All Fields]) OR "mask"[All Fields]) OR "N95"[All Fields]) OR "personal protective equipment"[All Fields])

FILTERS: randomized clinical trial; meta-analyses; systematic review; clinical trial

Pubmed search strategy (24/08/2020): 413 titles

((((((((((((("disinfect"[All Fields] OR "disinfectable"[All Fields]) OR "disinfectants"[Pharmacological Action]) OR "disinfectants"[MeSH Terms]) OR "disinfectants"[All Fields]) OR "disinfectant"[All Fields]) OR "disinfected"[All Fields]) OR "disinfecting"[All Fields]) OR "disinfection"[MeSH Terms]) OR "disinfection"[All Fields]) OR "disinfections"[All Fields]) OR "disinfective"[All Fields]) OR "disinfects"[All Fields]) AND (((((("virology"[MeSH Subheading] OR "virology"[All Fields]) OR "viruses"[All Fields]) OR "viruses"[MeSH Terms]) OR "virus s"[All Fields]) OR "viruse"[All Fields]) OR "virus"[All Fields])) AND (((((((("carrier state"[MeSH Terms] OR ("carrier"[All Fields] AND "state"[All Fields])) OR "carrier state"[All Fields]) OR "carrier"[All Fields]) OR "carrier s"[All Fields]) OR "heterozygote"[MeSH Terms]) OR "heterozygote"[All Fields]) OR "carriers"[All Fields]) OR ((("suspension s"[All Fields] OR "suspensions"[MeSH Terms]) OR "suspensions"[All Fields]) OR "suspension"[All Fields]))

Embase search strategy (24/08/2020): 310 titles

('viruses'/exp OR viruses OR 'influenza'/exp OR influenza OR 'airborne pathogens') AND ('disinfection'/exp OR disinfection OR 'disinfectant'/exp OR disinfectant OR 'biocidal agents' OR 'disinfection agents') AND ([systematic review]/lim OR [meta analysis]/lim OR [randomized controlled trial]/lim)

Embase search strategy (24/08/2020): 108 titles

('viruses'/exp OR viruses OR 'influenza'/exp OR influenza OR 'airborne pathogens') AND ('mask'/exp OR mask OR n95 OR 'personal protective equipment'/exp OR 'personal protective equipment') AND ([meta analysis]/lim OR [randomized controlled trial]/lim)

Embase search strategy (24/08/2020): 261 titles

('disinfection'/exp OR disinfection) AND ('viruses'/exp OR viruses) AND ('carrier'/exp OR carrier OR 'suspension'/exp OR suspension)

Cochrane Central Register of Controlled Trials search strategy (24/08/2020): 67 trials

(viruses OR influenza OR ‘airborne pathogens’) AND (disinfection OR disinfectant OR ‘biocidal agents’)

Cochrane Central Register of Controlled Trials search strategy (24/08/2020): 77 trials

(viruses OR influenza OR ‘airborne pathogens’) AND (mask OR N95 OR ‘Personal protective equipment’)

***Studies selection and Data collection process***

*SARS-Cov-2 and dentistry*

Studies reporting direct evidence about the efficacy of SARS-CoV-2 infected surfaces decontamination and about the efficacy of personal protective equipment (PPE) in preventing infection were considered. Only manuscripts with English language with no restriction for publication type were included. Unfortunately, strict eligibility criteria could not be used for this SR. Title and abstract were firstly screened. Then studies meeting the eligibility criteria and/or published in dental journals were considered for full text evaluation. This search did not yield studies on SARS-Cov-2 in dentistry

*Other respiratory viruses*

Title and abstract were firstly screened. Then studies meeting the eligibility criteria were considered for full text evaluation. All the studies comparing the efficacy of different disinfection agents on inanimate surfaces in terms of viral load reduction/inactivation were considered. Randomized clinical trials comparing the efficacy of different protective masks in preventing respiratory infection were included also.

The references of previous SR and included studies were checked also for additional titles. Only English language manuscripts were included.
