# Supplementary figures and images for: Surface disinfection and protective masks for SARS‐CoV‐2 and other respiratory viruses: A review by SIdP COVID‐19 task force
Source: Oral Dis. 2020 Oct 6:10.1111/odi.13646. Online ahead of print. doi: 10.1111/odi.13646 (PMC7646272; doi:10.1111/odi.13646)

**Appendix 2: PRISMA flow diagrams for studies selection for SARS-Cov-2 and dentistry**


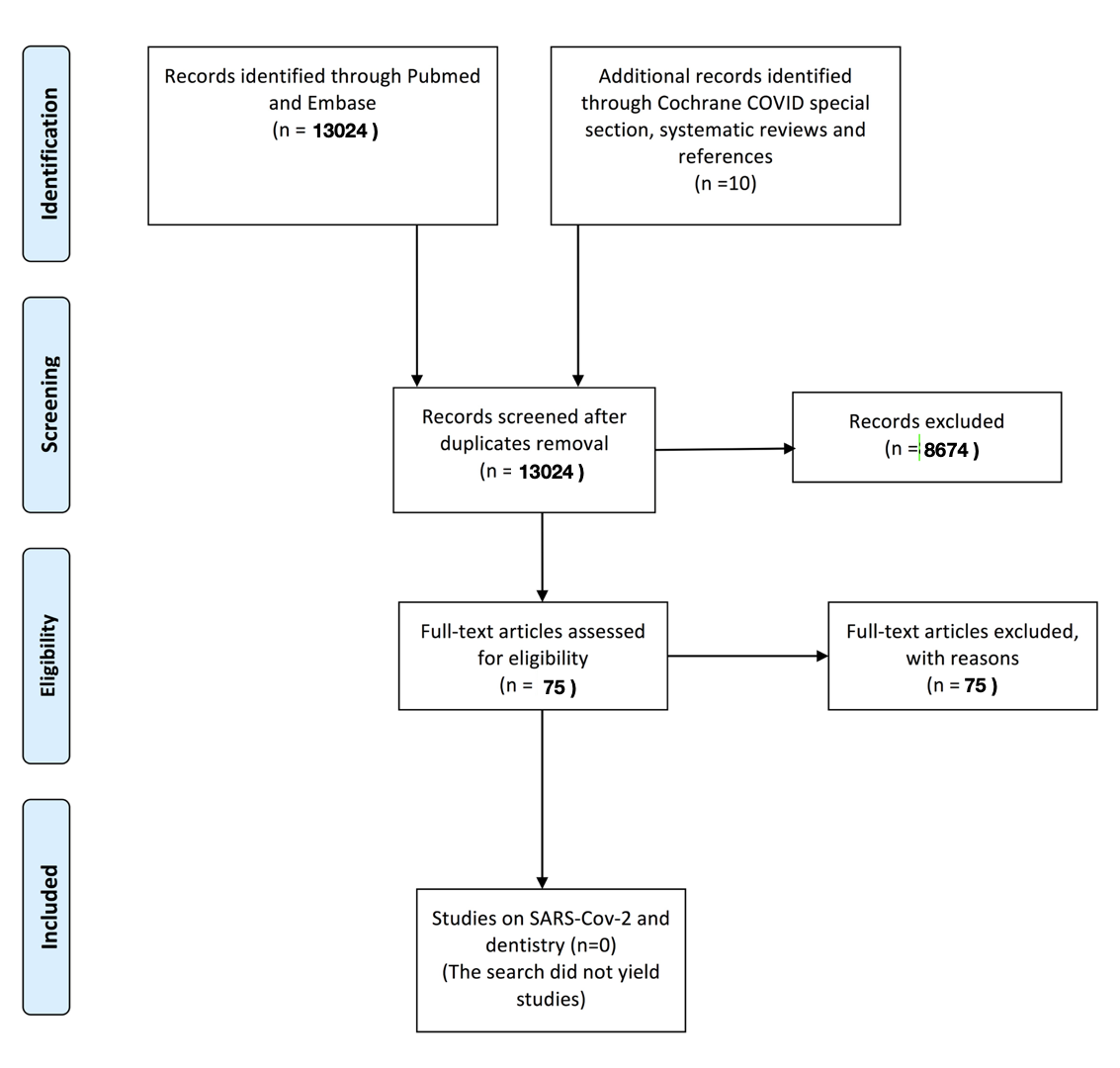

Supplement: Supplementary file 2 — Appendix S2 [file ODI-9999-0-s003.docx]

**Appendix 4:** PRISMA flow diagrams for studies selection for other viruses.


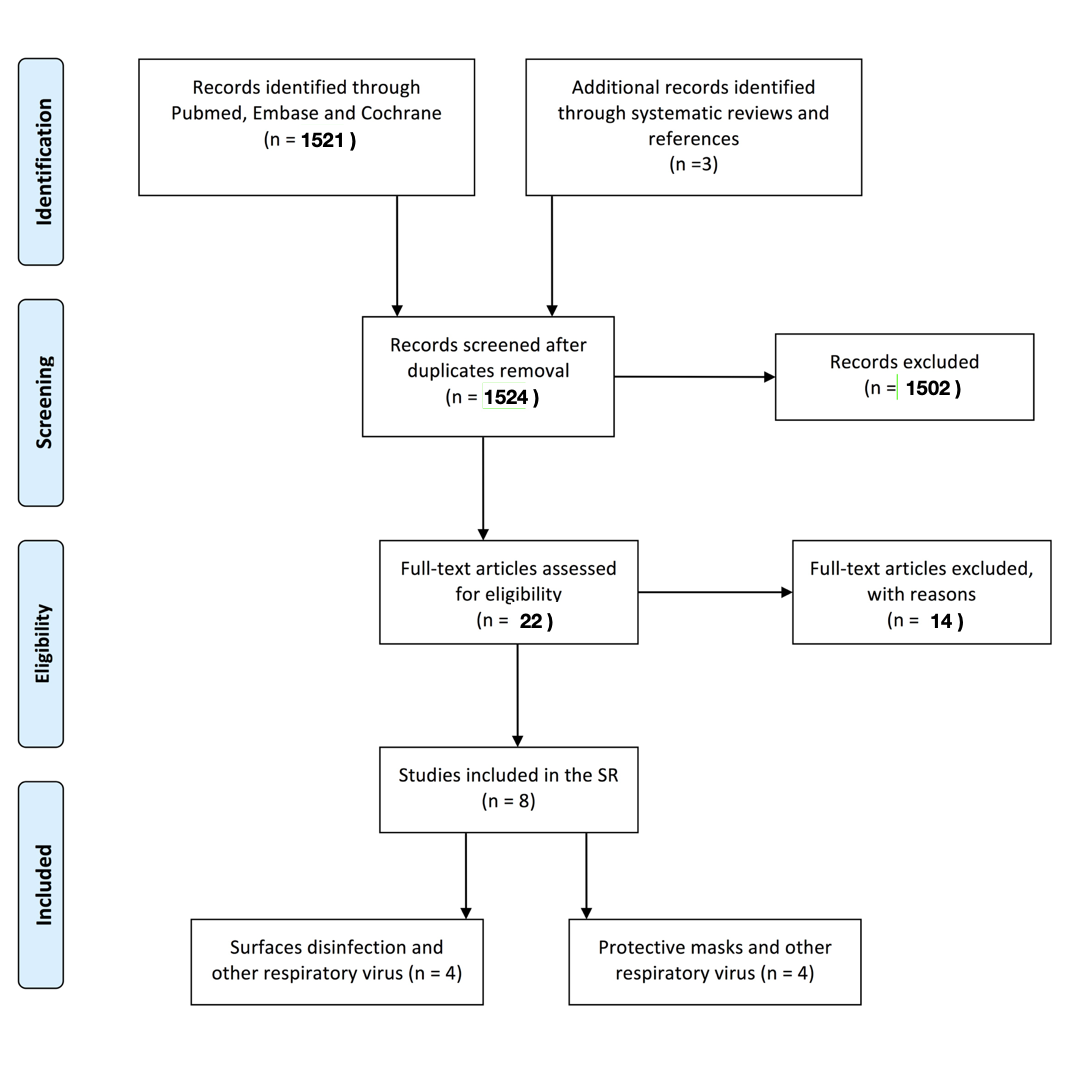

Supplement: Supplementary file 4 — Appendix S4 [file ODI-9999-0-s002.docx]
