# Supplementary material for: Surface disinfection and protective masks for SARS‐CoV‐2 and other respiratory viruses: A review by SIdP COVID‐19 task force
Source: Oral Dis. 2020 Oct 6:10.1111/odi.13646. Online ahead of print. doi: 10.1111/odi.13646 (PMC7646272; doi:10.1111/odi.13646)
Supplement: Supplementary file 3 — Appendix S3 [file ODI-9999-0-s005.docx]

**Appendix 3**: Table of excluded studies and related reasons for review SARS-Cov-2 and dentistry

| Ahmed et al. 2020; Almofada et al. 2020; Cagetti et al. 2020; De Stefani et al. 2020; Izzetti et al. 2020b; Khader et al. 2020; Kamate et al. 2020; Martina et al. 2020; Peloso et al. 2020; Putrino et al. 2020; Schacham et al. 2020; Sinjari et al 2020; Stangvaltaite-Mouhat et al. 2020 | Cross-sectional (questionnaire to assess knowledge, attitude and practices of dentists or patients) |
| --- | --- |
| Ather et al. 2020; Arduino et al. 2020; Berlin-Broner & Levin 2020; Braz-Silva et al. 2020; Cantore & Ballini 2020; Carrouel et al. 2020; Coulthard 2020; da Cruz Perez et al. 2020; Dave et al. 2020a; Dave et al. 2020b; Dziedzic & Wojtyczka 2020; Farooq & Ali 2020; Farshidfar & Hamedani 2020; Georgakopoulou 2020; Guo H. et al 2020; Guo Y et al. 2020; Halepas & Ferneini 2020; Lavigne et al. 2020; Lee & Auh 2020; Li et al. 2020; Mallineni et al 2020; Maret et al. 2020; Martelli-Júnior et al. 2020; Martìn Carreras-Presas et al. 2020; Mupparapu 2020; Odeh et al. 2020; Ortega et al. 2020; Prati et al. 2020; Sabino-Silva et al. 2020; Sardella et al. 2020; Sharma et al. 2020; Spagnuolo et al. 2020; Tarakji & Nassani 2020; Villa et al. 2020; Vinayachandran & Balasubramanian 2020a; Vinayachandran & Balasubramanian 2020b; Wu & Chang 2020 | Letter to Editor; Editorial; Commentary; Expert Opinion; Short communication |
| Abramovitz et al. 2020; Alharbi et al. 2020; Diegritz et al. 2020; Fallahi et al. 2020; Fiorillo et al. 2020; Gurzawska-Comis et al. 2020; Ilhan et al. 2020; Izzetti et al. 2020b; Iyer et al. 2020; Jamal et al 2020; Lo Giudice 2020; Lucaciu et al. 2020; Pan et al. 2020; Ren et al. 2020; Sa et al. 2020; Volgenant et al. 2020; Villani et al. 2020 | Narrative review, Clinical recommendation |
| Farronato et al. 2020; Peng et al. 2020; Umer et al. 2020; Xu et al. 2020 | Review on a specific issue |
| Meng et al. 2020 | Review and clinical experience |
| Lamas et al. 2020; Long & Corsar 2020; Yu et al. 2020 | Original research |

***References of excluded studies***

1. Abramovitz, I., Palmon, A., Levy, D., Karabucak, B., Kot-Limon, N., Shay, B., … Almoznino, G. (2020). Dental care during the coronavirus disease 2019 (COVID-19) outbreak: operatory considerations and clinical aspects. Quintessence Int, 51(5):418-429. doi: 10.3290/j.qi.a44392.
2. Ahmed, M.A., Jouhar, R., Ahmed, N., Adnan, S., Aftab, M., Zafar, M.S., Khurshid, Z. (2020) Fear and Practice Modifications among Dentists to Combat Novel Coronavirus Disease (COVID-19) Outbreak. Int J Environ Res Public Health, Apr 19;17(8):E2821. doi: 10.3390/ijerph17082821.
3. Alharbi, A., Alharbi, S., Alqaidi, S. (2020). Guidelines for dental care provision during the COVID-19 pandemic. Saudi Dent J, Apr 7. doi: 10.1016/j.sdentj.2020.04.001. [Epub ahead of print]
4. Almofada, S.K., Alherbisch, R.J., Almuhraj, N.A., Almeshary, B.N., Alrabiah, B., … Baseer,, M.A. (2020). Knowledge, Attitudes, and Practices Toward COVID-19 in a Saudi Arabian Population: A Cross-Sectional Study. Cureus. Jun 29,12(6),e8905. doi: 10.7759/cureus.8905
5. Arduino, P.G., Conrotto, D., Broccoletti, R. (2020) The outbreak of Novel Coronavirus disease (COVID-19) caused a worrying delay in the diagnosis of oral cancer in north-west Italy: the Turin Metropolitan Area experience. Oral Dis, Apr 19. doi: 10.1111/odi.13362. [Epub ahead of print]
6. Ather A., Patel B., Ruparel N.B., Diogenes A., Hargreaves K.M. (2020). Coronavirus Disease 19 (COVID-19): Implications for Clinical Dental Care. J Endod, Apr 6. doi: 10.1016/j.joen.2020.03.008. [Epub ahead of print]
7. Berlin-Broner, Y. & Levin, L. (2020). 'Dental Hierarchy of Needs' in the COVID-19 Era - or Why Treat When It Doesn't Hurt? Oral Health Prev Dent. 2020;18(2):95.
8. Braz-Silva P.H., Pallos D., Giannecchini, S., To K.K.W. (2020). SARS-CoV-2: What Can Saliva Tell Us? Oral Diseases, May. doi: 10.1111/ODI.13365
9. Cagetti, M.G., Cairoli, J.L., Senna, A., Campus, G. (2020). COVID-19 Outbreak in North Italy: An Overview on Dentistry. A Questionnaire. Int J Environ Res Public Health, May 28, 17(11), E3835. doi:10.3390/ijerph17113835.
10. Cantore, S. & Ballini, A. (2020). Coronavirus disease 2019 (COVID-19) pandemic burst and its relevant consequences in dental practice. Open Dentistry Journal, 14:1 (111-112). Date of Publication: 2020. DOI: 10.2174/1874210602014010111.
11. Carrouel, F., Conte, M.P., Fisher, J., Gonçalves, L.S., Dussart, C., Llodra, J.C., Bourgeois, D. (2020). COVID-19: A recommendation to examine the effect of mouthrinses with β-cyclodextrin combined with citrox in preventing infection and progression. Journal of Clinical Medicine, 9:4 Article Number: 1126. doi:10.3390/jcm9041126
12. Coulthard, P. (2020) Dentistry and coronavirus (COVID-19) - moral decision-making. Br Dent J. 2020 Apr;228(7):503-505. doi: 10.1038/s41415-020-1482-1.
13. da Cruz Perez, D.E., Passos, K.K.M., Machado, R.A., Martelli-Junior, H., Bonan, P.R.F. (2020) Continuing education in oral cancer during coronavirus disease 2019 (covid-19) outbreak Oral Oncology. Article Number: 104713. Date of Publication: 2020 doi:10.1016/j.oraloncology.2020.104713
14. Dave, M., Coulthard, P., Patel N., Seoudi, N., Horner K. (2020a) Letter to the Editor: Use of Dental Radiography in the COVID-19 Pandemic. J Dent Res, Apr 23:22034520923323. doi: 10.1177/0022034520923323. Online ahead of print.
15. Dave, M., Seoudi, N., Coulthard, P. (2020b). Urgent dental care for patients during the COVID-19 pandemic. Lancet, Apr 18;395, 1257. doi: 10.1016/S0140-6736(20)30806-0. Epub 2020 Apr 3.
16. De Stefani, A., Bruno, G., Mutinelli, S., Gracco, A. (2020). COVID-19 Outbreak Perception in Italian Dentists. Int J Environ Res Public Health, May 29, 17(11), E3867. doi:10.3390/ijerph17113867
17. Diegritz, C., Manhart, J., Bucher, K., Grabein, B., Schuierer, G., …Fotiadou, C. (2020) A detailed report on the measures taken in the Department of Conservative Dentistry and Periodontology in Munich at the beginning of the COVID-19 outbreak. Clin Oral Investig Aug;24(8),2931-2941. doi: 10.1007/s00784-020-03440-z. Epub 2020 Jul 1.
18. Dziedzic, A. & Wojtyczka, R. (2020). The impact of coronavirus infectious disease 19 (COVID-19) on oral health. Oral Dis, Apr 18. doi: 10.1111/odi.13359
19. Fallahi, H.R., Keyhan, S.O., Zandian, D., Kim, S.G., Cheshmi, B. (2020) Being a front-line dentist during the Covid-19 pandemic: a literature review. Maxillofac Plast Reconstr Surg, Apr 24;42(1):12. doi: 10.1186/s40902-020-00256-5. eCollection 2020 Dec.
20. Farooq, I. & Ali, S. (2020). COVID-19 outbreak and its monetary implications for dental practices, hospitals and healthcare workers Postgraduate Medical Journal, Article Number: 137781. Date of Publication: 2020 doi: 10.1136/postgradmedj-2020-137781
21. Farronato, M., Boccalari, E., Del Rosso, E., Lanteri, V., Mulder, R., Maspero, C. (2020). A Scoping Review of Respirator Literature and a Survey among Dental Professionals. Int J Environ Res Public Health, Aug 17,17(16),E5968. doi: 10.3390/ijerph17165968
22. Farshidfar, N., & Hamedani, S. (2020). Hyposalivation as a potential risk for SARS-CoV-2 infection: Inhibitory role of saliva. Oral Dis. 2020 Apr 29. doi: 10.1111/ odi.13375.
23. Fiorillo, L., Cervino, G., Matarese, M., D'Amico, C., Surace, G., Paduano, V., … Cicciù, M. (2020). COVID-19 Surface Persistence: A Recent Data Summary and Its Importance for Medical and Dental Settings. Int J Environ Res Public Health, Apr 30;17(9):E3132. doi: 10.3390/ijerph17093132.
24. Georgakopoulou, E.A. (2020). Digitally aided telemedicine during the SARS-CoV-2 pandemic to screen oral medicine emergencies. Oral Dis, May 5. doi: 10.1111/odi.13383. Online ahead of print.
25. Guo, H., Zhou, Y., Liu, X., Tan, J. (2020). The impact of the COVID-19 epidemic on the utilization of emergency dental services. J Dent Sci, Mar 16. doi: 10.1016/j.jds.2020.02.002. [Epub ahead of print]
26. Guo, Y., Yuan, C., Wei, C. (2020). Emergency Measures for Acute Oral Mucosa Diseases During the Outbreak of COVID-19. Oral Dis, Apr 11. doi: 10.1111/odi.13350
27. Gurzawska-Comis, K., Becker, K., Brunello, G., Gurzawska, A., Schwarz F. (2020). Recommendations for Dental Care during COVID-19 Pandemic. J Clin Med, Jun 12, 9(6), E1833. doi: 10.3390/jcm9061833
28. Halepas, S. & Ferneini, E.M. (2020). A Pinch of Prevention is Worth a Pound of Cure: Proactive Dentistry in the Wake of COVID-19. J Oral Maxillofac Surg, Apr 9. doi: 10.1016/j.joms.2020.03.036. [Epub ahead of print]
29. Ilhan, B., Bayrakdar, I.S., Orhan, K. (2020). Dental radiographic procedures during COVID-19 outbreak and normalization period: recommendations on infection control. Oral Radiol, Jun 29;1-5. doi: 10.1007/s11282-020-00460-z
30. Iyer, P., Aziz, K., Ojcius, D.M. (2020) Impact of COVID-19 on dental education in the United States. J Dent Educ, Apr 27. doi: 10.1002/jdd.12163.
31. Izzetti, R., Nisi M., Gabriele M., Graziani F. (2020a). COVID-19 Transmission in Dental Practice: Brief Review of Preventive Measures in Italy. J Dent Res, Apr. doi:10.1177/0022034520920580. [Epubahead of print]
32. Izzetti, R., Gennai, S., Nisi M., Barone, A., Giuca, M.R., … Graziani F. (2020b). A perspective on dental activity during COVID-19: the Itailan Survey. Oral Dis. Aug 13;10.1111/odi.13606. doi: 10.1111/odi.13606
33. Jamal, M., Shah, M., Almarzooqi, S.H., Aber, H., Khawaja, S., El Abed, R., … Samaranayake, L.P. (2020). Overview of transnational recommendations for COVID‐19 transmission control in dental care settings. Oral dis, 19 May. doi.org/10.1111/odi.13431
34. Kamate, S.K., Sharma, S., Thakar, S., Srivastava, D., Sengupta, K., Hadi, A.J., … Dhanker, K. (2020). Assessing Knowledge, Attitudes and Practices of dental practitioners regarding the COVID-19 pandemic: A multinational study. *Dent Med Probl* 57(1), 11–17. doi:10.17219/dmp/119743
35. Khader Y., Al Nsour M., Al-Batayneh O.B., Saadeh R., Bashier H., Alfaqih M, Al-Azzam S., AlShurman B.A. (2020). Dentists' Awareness, Perception, and Attitude Regarding COVID-19 and Infection Control: Cross-Sectional Study Among Jordanian Dentists. JMIR Public Health Surveill, Apr 9;6(2):e18798. doi: 10.2196/18798
36. Martina, S., Amato, A., Rongo, R., Caggiano, M., Amato, M. (2020). The perception of COVID-19 Among Italian Dentists: an orthodontic point of view. Int J Environ Res Public Health, Jun 18, 17(12), E4384. doi:10.3390/ijerph17124384
37. Lamas, M.L., Dios, P.D., Perez Rodrìguez, M.T., Del Campo Pérez, V., Cabrera Alvargonzalez, J.J., Lòpez Domìnguez, A.M., … Limeres Posse, J. (2020). Is povidone iodine mouthwash effective against SARS‐CoV‐2? First in vivo tests. Oral Dis, doi.org/10.1111/odi.13526
38. Lavigne, G., Fabbro, C.D., Babiloni, A.H., Huynh, N., Gauthier, L., Arcache, P., Masse, J.F. (2020). Dental sleep medicine perspectives post-COVID-19: interprofessional adaptation and directions. J Clin Sleep Med, May 4. doi: 10.5664/jcsm.8546. Online ahead of print.
39. Lee, YH & Auh Q-Schick. (2020). Strategies for prevention of coronavirus Disease 2019 in the dental field. Oral Dis, 2020 Apr 19. doi: 10.1111/odi.13361
40. Li, D.T.S., Samaranayake, L.P., Leung, Y.Y., Neelakantan, P. (2020). Facial protection in the era of COVID-19: a narrative review. Oral Dis, Jun 7. doi: 10.1111/odi.13460
41. Lo Giudice, R. (2020). The Severe Acute Respiratory Syndrome Coronavirus-2 (SARS CoV-2) in Dentistry. Management of Biological Risk in Dental Practice. Int J Environ Res Public Health, Apr 28;17(9),E3067. doi:10.3390/ijerph17093067.
42. Long, L. & Corsar, K. (2020). The COVID-19 effect: number of patients presenting to The Mid Yorkshire Hospitals OMFS team with dental infections before and during The COVID-19 outbreak. Br J Oral Maxillofac Surg. 2020 May 1:S0266-4356(20)30184-4. doi:10.1016/j.bjoms.2020.04.030
43. Lucaciu, O., Tarczali, D., Petrescu, N. (2020). Oral healthcare during the COVID-19 pandemic, Journal of Dental Sciences, https://doi.org/ 10.1016/j.jds.2020.04.012
44. Mallineni, S.K., Innes, N.P., Raggio, D.P., Araujo, M.P., Robertson, M.D., Jayaraman, J. (2020) Coronavirus disease (COVID-19): Characteristics in children and considerations for dentists providing their care. Int J Paediatr Dent, Apr 6. doi: 10.1111/ipd.12653. [Epub ahead of print]
45. Maret, D., Peters, O.A., Vaysse, F., Vigarios, E. (2020). Integration of telemedicine into the public health response to COVID-19 must include dentists. Int Endod J, Apr 22. doi: 10.1111/iej.13312. Online ahead of print.
46. Martelli-Júnior, H., Machado, R.A., Martelli, D.R.B., Coletta, R.D.(2020). Dental journals and coronavirus disease (COVID-19): A current view. Oral Oncol, Apr 2:104664. doi: 10.1016/j.oraloncology.2020.104664. [Epubahead of print]
47. Martín Carreras-Presas, C., Amaro Sánchez, J., López-Sánchez, A.F., Jané-Salas, E., Somacarrera Pérez, M.L. (2020). Oral vesiculobullous lesions associated with SARS-CoV-2 infection. Oral Dis, May 5. doi: 10.1111/odi.13382. Online ahead of print.
48. Meng, L., Hua, F., Bian, Z. (2020). Coronavirus Disease 2019 (COVID-19): Emerging and Future Challenges for Dental and Oral Medicine. J Dent Res. 2020 May;99(5):481-487. doi: 10.1177/0022034520914246. Epub 2020 Mar 12.
49. Mupparapu, M. (2020) Editorial: Dental practitioners' role in the assessment and containment of coronavirus disease (COVID-19): Evolving recommendations from the Centers for Disease Control. Quintessence Int. 2020;51(5):349-350. doi: 10.3290/j.qi.a44446.
50. Odeh, N.D., Babkair, H., Abu-Hammad, S., Borzangy, S., Abu-Hammad, A., Abu-Hammad, O. (2020). COVID-19: Present and Future Challenges for Dental Practice. Int J Environ Res Public Health, Apr 30;17(9):E3151. doi:10.3390/ijerph17093151.
51. Ortega, K.L., de Oliveira Rech, B., Ferreira Costa, A.L., Pérez Sayáns, M., Braz-Silva, P.H. (2020). Is 0.5% Hydrogen Peroxide Effective against SARS-CoV-2? Oral Dis, Jun 21. doi: 10.1111/odi.13503
52. Pan, Y., Liu, H., Chu, C., Li, X., Liu, S., Lu, S. (2020). Transmission routes of SARS-CoV-2 and protective measures in dental clinics during the COVID-19 pandemic. Am J Dent, Jun 33(3), 129-134.
53. Peloso, R.M., Pavesi Pini, N.I., Neto, D.S., Mori, A.A., Gobbi de Oliveira, R.C., … Freitas, K.M.S. (2020). How does the quarantine resulting from COVID-19 impact dental appointments and patient anxiety levels? Braz Oral Res Jun 29;34:e84. doi: 10.1590/1807-3107bor-2020.vol34.0084. eCollection 2020
54. Peng, X., Xu X., Li Y., Cheng L., Zhou X., Ren B. (2020). Transmission routes of 2019-nCoV and controls in dental practice. Int J Oral Sci, Mar 3, 12(1), 9. doi: 10.1038/s41368-020-0075-9.
55. Prati, C., Pelliccioni, G.A., Sambri, V., Chersoni, S., Gandolfi, M.G. (2020). COVID-19: its impact on dental schools in Italy, clinical problems in endodontic therapy and general considerations. Int Endod J, May 53(5),723-725. doi: 10.1111/iej.13291.
56. Putrino, A., Raso, M., Magazzino, C., Galluccio, G. (2020). Coronavirus (COVID-19) in Italy: knowledge, management of patients and clinical experience of Italian dentists during the spread of contagion. BMC Oral Health, Jul 10;20(1):200. doi: 10.1186/s12903-020-01187-3.
57. Ren, Y.F., Rasubala, L., Malmstrom, H., Eliav, E. (2020). Dental Care and Oral Health under the Clouds of COVID-19. JDR Clin Trans Res, Apr 24. doi:10.1177/2380084420924385. Online ahead of print.
58. Sa, Y., Lin, W-S., Morton, D., Huang, C. (2020). Coronavirus disease 2019 (COVID-19): Experiences and protocols from the Department of Prosthodontics at the Wuhan University. J Prosthet Dent, Jul 10;S0022-3913(20)30374-7. doi: 10.1016/j.prosdent.2020.06.004
59. Sabino-Silva, R., Jardim, A.C.G., Siqueira, W.L. (2020). Coronavirus COVID-19 impacts to dentistry and potential salivary diagnosis. Clinical oral investigations, 24:4, (1619-1621). Date of Publication: 1 Apr 2020 doi: 10.1007/s00784-020-03248-x
60. Sardella, A., Varoni, E., Carrassi, A., Pispero, A., Lombardi, N., Lodi, G. (2020). Who's afraid of the big bad wolf? The experience of an Oral Medicine Unit in the time of Corona-Virus. Oral Dis, Jun 7. doi: 10.1111/odi.13461
61. Shacham, M., Hamama-Raz, Y., Kolerman, R., Mijiritsky, O., Ben-Ezra, M., Mijiritsky, E. (2020). COVID-19 Factors and Psychological Factors Associated with Elevated Psychological Distress among Dentists and Dental Hygienists in Israel. Int J Environ Res Public Health, Apr 22;17(8):E2900. doi: 10.3390/ijerph17082900.
62. Sharma, S., Kumar, V., Chawla, A., Logani, A. (2020) Rapid detection of SARS-CoV-2 in saliva: Can an endodontist take the lead in point-of-care COVID-19 testing? Int Endod J, Apr 28. doi: 10.1111/iej.13317. Online ahead of print.
63. Sinjari, B., Rexhepi, I., Santilli, M., D’Addazio, G., Chiacchiaretta, P., Di Carlo, P., Caputi, S. (2020). The Impact of COVID-19 Related Lockdown on Dental Practice in Central Italy-Outcomes of A Survey. Int J Environ Res Pubblic Health, Aug 10,17(16),E5780. doi: 10.3390/ijerph17165780
64. Spagnuolo, G., De Vito, D., Rengo, S., Tatullo, M. (2020). COVID-19 Outbreak: An Overview on Dentistry. Int J Environ Res Public Health, Mar 22;17(6). doi:10.3390/ijerph17062094.
65. Stangvaltaite-Mouhat, L., Uhlen, M-M., Skudutyte-Rysstad, R., Hovden, E.A.S., Shabestari, M., Ansteinsson, V.E. (2020). Dental Health Services Response to COVID-19 in Norway. Int J Environ Res Public Health Aug 12,17(16),E5843. doi: 10.3390/ijerph17165843
66. Tarakji, B. & Nassani, M.Z. (2020). Reactivation of COVID-19 - 14 days from the onset of symptoms may not be enough to allow dental treatment. Oral Dis, Jun 10. doi: 10.1111/odi.13487
67. Umer, F., Haji, Z., Zafar, K. (2020). Role of respirators in controlling the spread of Novel Coronavirus (Covid-19) among dental health care providers: a review. Int Endod J, May 1. doi: 10.1111/iej.13313. Online ahead of print.
68. Volgenant, C.M.C., Persoon, I.F., de Ruijter R.A.G., de Soet, J.J.H. (2020). Infection control in dental health care during and after the SARS‐CoV‐2 outbreak. Oral dis, 11 may. doi.org/10.1111/odi.13408
69. Villa, A., Sankar, V., Shiboski, C. (2020). Tele(oral)medicine: a new approach during the COVID-19 crisis. Oral Dis, Apr 20. doi: 10.1111/odi.13364. [Epub ahead of print]
70. Villani, F.A., Aiuto, R., Paglia, L., Re, D. (2020) COVID-19 and Dentistry: prevention in dental practice, a literature review. Int J environ Res Public Health Jun 26;17(12):4609. doi: 10.3390/ijerph17124609.
71. Vinayachandran, D., Balasubramanian, S. (2020a). Is Gustatory Impairment the First Report of an Oral Manifestation in COVID-19? Oral Dis, Apr 25. doi: 10.1111/odi.13371. Online ahead of print.
72. Vinayachandran, D., Balasubramanian, S. (2020b) Salivary diagnostics in COVID-19: Future research implications. J Dent Sci, Apr 23. doi: 10.1016/j.jds.2020.04.006. Online ahead of print.
73. Wu, M., & Chang, Y.C. (2020). COVID-19 and its implications in the management of resource infrastructure. J Dent Sci, Apr 26. doi: 10.1016/j.jds.2020.04.008. Online ahead of print.
74. Xu, R., Cui, B., Duan, X., Zhang, P., Zhou, X., Yuan, Q. (2020). Saliva: potential diagnostic value and transmission of 2019-nCoV. Int J Oral Sci, Apr 17, 12(1):11. doi: 10.1038/s41368-020-0080-z.
75. Yu, J., Zhang, T., Zhao, D., Haapasalo, M., Shen, Y.(2020). Characteristics of Endodontic Emergencies during Coronavirus Disease 2019 Outbreak in Wuhan. J Endod, Apr 10:S0099-2399(20)30238-7. doi: 10.1016/j.joen.2020.04.001.
