# Supplementary material for: Surface disinfection and protective masks for SARS‐CoV‐2 and other respiratory viruses: A review by SIdP COVID‐19 task force
Source: Oral Dis. 2020 Oct 6:10.1111/odi.13646. Online ahead of print. doi: 10.1111/odi.13646 (PMC7646272; doi:10.1111/odi.13646)
Supplement: Supplementary file 5 — Appendix S5 [file ODI-9999-0-s001.docx]

**Appendix 5**: Table of excluded studies and related reasons for review on other respiratory viruses.

| Hulkower et al. 2011; Lemmer et al. 2017: Orel et al. 2020 | Not respiratory virus |
| --- | --- |
| Tuladhar et al. 2015 | Study on hand disinfection |
| Tuladhar et al. 2012 | Study on Hydrogen peroxide vapour |
| Rabenau et al. 2005 | Study on the stability of SARS-Cov under different conditions |
| Aiello et al. 2012; Barasheed et al. 2011; MacIntyre et al. 2016; Wang et al. 2015; | Participants were not HCW |
| Bishoff et al. 2017 | Laboratory testing |
| Jacobs et al.2009 | Study not comparing different type of masks |
| MacIntyre 2015 | Study on cloth mask |
| MacIntyre 2017 | This study pooled data from previously published RCTs |

***References of excluded studies***

1. Aiello, A.E., Perez, V., Coulborn, R.M., Davis, B.M., Uddin, M., Monto. A.S. (2012). Facemasks, hand hygiene, and influenza among young adults: A randomized intervention trial. PLoS ONE, 7:1 Article Number: e29744.
2. Barasheed, O., Almasri, N., Badahdah, A.M., Heron, L., Taylor, J., … Booy, R. (2011). Pilot Randomised Controlled Trial to Test Effectiveness of Facemasks in Preventing Influenza-like Illness Transmission among Australian Hajj Pilgrims in Infect Disord Drug Targets, 14(2), 110-6. doi: 10.2174/1871526514666141021112855.
3. Bischoff, W., Turner, J., Russell, G.B., Blevins, M.; Stehle, J. (2017). Evaluation of a novel powered air-purifying respirator (PAPR) vs. a N95 respirator mask for the protection against influenza in a human exposure model. Open Forum Infectious Diseases, 4 Supplement 1 (S168).
4. Hulkower, R.L., Casanova, L.M., Rutala, W.A., Weber, D.J., Sobsey, M.D. (2011). Inactivation of Surrogate Coronaviruses on Hard Surfaces by Health Care Germicides. Am J Infect Control, Jun 39(5),401-407. doi: 10.1016/j.ajic.2010.08.011
5. Jacobs, J.L., Ohde, S., Takahashi, O., Tokuda, Y., Omata, F., Fukui, T. (2009). Use of surgical face masks to reduce the incidence of the common cold among health care workers in Japan: A randomized controlled trial. American Journal of Infection Control, 37:5, 417-419.
6. Lemmer, K., Howaldt, S., Heinrich, R., Roder, A., Pauli, G,… Grunow, R. (2017). Test Methods for Estimating the Efficacy of the Fast-Acting Disinfectant Peracetic Acid on Surfaces of Personal Protective Equipment. J Appl Microbiol, Nov 123(5), 1168-1183. doi: 10.1111/jam.13575
7. MacIntyre, C.R., Chughtai, A.A., Rahman, B., Peng, Y., Zhang, Y., … Wang Q. (2017). The efficacy of medical masks and respirators against respiratory infection in healthcare workers. Influenza Other Respir Viruses, Nov;11(6), 511-517. doi: 10.1111/irv.12474.
8. MacIntyre, C.R., Seale, H., Dung, T.C., Hien, N.T., Nga, P.T., … Wang Q. (2015). A cluster randomised trial of cloth masks compared with medical masks in healthcare workers. BMJ Open Apr 22;5(4):e006577. doi: 10.1136/bmjopen-2014-006577.
9. MacIntyre, C.R., Zhang, Y., Chughtai, A.A., Seale, H., Zhang, D., … Wang, Q. (2016). Cluster randomised controlled trial to examine medical mask use as source control for people with respiratory illness. BMJ Open, Dec 30;6(12):e012330. doi: 10.1136/bmjopen-2016-012330.
10. Orel, I., Graf, H., Riou, P. (2020). Decontamination efficacy of sodium hypochlorite solution for poliovirus. Biologicals, Aug 14;S1045-1056(20)30087-7. doi: 10.1016/j.biologicals.2020.07.007
11. Rabenau, H.F., Cinatl, J., Margenstern, B., Bauer, G., Preiser, W., Doerr, H.W. (2005). Stabiliy and inactivation of SARS coronavirus, Jan 194 (1-2), 1-6. Doi:10.1007/s00430-004-0219-0.
12. Tuladhar, E., Hazeleger, W.C., Koompmans, M., Zwietering, M.H., Duizer, E., Beumer, R.R. (2015). Reducing Viral Contamination From Finger Pads: Handwashing Is More Effective Than Alcohol-Based Hand Disinfectants. J Hosp Infect, Jul;90(3):226-34. doi:10.1016/j.jhin.2015.02.019
13. Tuladhar, E., Terpstra, P., Koompmans, M., Duizer, E. (2012). Virucidal Efficacy of Hydrogen Peroxide Vapour Disinfection. J Hosp Infect, Feb 80(2),110-5. doi: 10.1016/j.jhin.2011.10.012.
14. Wang, M., Barasheed, O., Rashid, H., Booy, R., El Bashir, H., … Heron, L.A. (2015). A cluster-randomised controlled trial to test the efficacy of facemasks in preventing respiratory viral infection among Hajj pilgrims..J Epidemiol Glob Health. Jun;5(2):181-9. doi:10.1016/j.jegh.2014.08
